# Sex-based differences in risk of revision for infection after hip, knee, shoulder, and ankle arthroplasty in osteoarthritis patients: a multinational registry study of 4,800,000 implants

**DOI:** 10.2340/17453674.2024.42183

**Published:** 2024-12-10

**Authors:** Anne M C ROERINK, Rob G H H NELISSEN, Carl HOLDER, Stephen E GRAVES, Michael DUNBAR, Eric BOHM, Alexander W GRIMBERG, Arnd STEINBRÜCK, Håvard DALE, Anne Marie FENSTAD, Ashley W BLOM, Erik LENGUERRAND, Christopher FRAMPTON, Tine WILLEMS, Jan VICTOR, Mireia ESPALLARGUES, Jorge ARIAS-DE LA TORRE, Enrico CIMINELLO, Marina TORRE, Bart G PIJLS

**Affiliations:** 1 Department of Orthopaedics, Leiden University Medical Center, Leiden, The Netherlands; 2 Department of Orthopaedics, Leiden University Medical Center, Leiden, The Netherlands; 3 South Australian Health and Medical Research Institute (SAHMRI), Adelaide, Australia; 4 Australian Orthopaedic Association National Joint Replacement Registry (AOANJRR), Adelaide, Australia; 5 Clinical and Health Sciences, University of South Australia, Adelaide, Australia; 6 Division of Orthopaedics, Dalhousie University, Halifax, Nova Scotia, Canada; 7 Canadian Joint Replacement Registry, Canada; 8 Concordia Joint Replacement Group, University of Manitoba, Winnipeg, Canada; 9 German Arthroplasty Registry (EPRD Deutsche Endoprothesenregister gGmbH), Berlin, Germany; 10 The Norwegian Arthroplasty Register, Department of Orthopedic Surgery, Haukeland University Hospital, Bergen, Norway; 11 Department of Clinical Medicine, University of Bergen, Bergen, Norway; 12 Bristol Medical School, University of Bristol, Bristol, UK; 13 Department of Medicine, University of Otago, Christchurch, New Zealand; 14 Department of Rehabilitation Sciences, Ghent University, Ghent, Belgium; 15 Department of Orthopedics and Traumatology; Ghent University, Ghent University Hospital, Ghent, Belgium; 16 Agència de Qualitat i Avaluació Sanitàries de Catalunya (AQuAS), Barcelona, Spain; 17 Care in Long Term Conditions Research Division, King’s College London, London, UK; 18 CIBER Epidemiology and Public Health (CIBERESP), Madrid, Spain; 19 Institute of Biomedicine (IBIOMED). Universidad de León, León, Spain; 20 Italian Arthroplasty Registry (RIAP), Rome, Italy; 21 Italian National Institute of Health, Rome, Italy; 22 Department of Orthopaedics, Leiden University Medical Center, Leiden, The Netherlands

## Abstract

**Background and purpose:**

We aimed to determine sex differences for periprosthetic joint infections after primary arthroplasty of the hip, knee, ankle, and shoulder in osteoarthritis patients in an international perspective.

**Methods:**

This is a multinational combined arthroplasty registry study. Each arthroplasty registry performed Cox-regression analysis of their data and reported the crude and adjusted hazard ratios (HR) with an a priori designed data form. A random-effects model was used to pool these HRs to estimate an overall HR with 95% confidence interval (CI). Adjustment was undertaken for patient age, BMI, ASA grade, type of fixation, and type of implant. 9 arthroplasty registries participated. Patients who received primary total joint arthroplasty for primary osteoarthritis were considered: 2,134,313 hip arthroplasties, 2,658,237 knee arthroplasties, 57,889 shoulder arthroplasties, and 8,445 ankle arthroplasties. We calculated hazard ratios (HR) for the overall risk of complete revision due to infection for each implant type and follow-up.

**Results:**

The pooled HR for revision due to infection for men compared with women at 1-year follow-up was 1.60 (95% confidence interval [CI] 1.42–1.80) for hip arthroplasties; 2.06 (CI 1.90–2.46) for knee arthroplasties; 4.51 (CI 2.99–6.80) for shoulder arthroplasties; and 0.87 (CI 0.46–1.62) for ankle arthroplasties. These results remained consistent over time and were identified in both unadjusted and adjusted models.

**Conclusion:**

Men have a higher risk of revision due to infection than women after primary hip, knee, and shoulder arthroplasty. No evidence of difference was found for ankle arthroplasty. These elevated relative risks persist in the fully adjusted investigations and over the 10-year postoperative period studied.

Periprosthetic joint infection (PJI) is a rare but devastating complication of arthroplasty occurring in 1–2% of primary and in 4% of revision arthroplasties [1,2]. PJIs have a profoundly negative physical and psychological impact on patients, with a protracted course usually involving prolonged treatment with antibiotics, multiple surgeries, uncertain outcomes, increased mortality, and high costs [3-5]. PJI has a serious impact on patients’ lives, but also on daily clinical practice. Its management is multifactorial and must be addressed in a multidisciplinary manner: choices and circumstances concerning the prosthesis and surgery play a significant role, as do patient factors and the state of their immune system [6].

Recent published studies have presented data showing sex-based differences in immune response resulting in differential sex susceptibility to autoimmune diseases, malignancies, outcome of vaccination, and infectious diseases. Males are generally more susceptible to infectious diseases [7]. Sex chromosomes, hormones, and environmental factors contribute to different regulation of the immune system [8]. Whereas estrogens largely enhance the immune response, androgens and progesterone mainly suppress it [9]. Future studies could focus on the association between sex hormones and risk of PJI.

Few studies on orthopedic surgery have evaluated both sexes separately, resulting in masked sex-specific effects on outcome [7,10,11]. Therefore, in this study we aim to answer the question: do men have a higher risk of revision for PJI after primary arthroplasty for osteoarthritis than women?

## Methods

This is a multinational combined arthroplasty registry study. The population of interest included all patients treated with primary hip, knee, shoulder, or ankle arthroplasty for primary osteoarthritis. Patients with revision or hemi-arthroplasties were excluded. To reduce possible confounding due to underlying comorbidities and immunosuppressive drugs, patients with total joint arthroplasties for secondary osteoarthritis were excluded. The outcome of interest was total revision for PJI (revision of all the components in a 1- or 2-stage setting), in which PJI was diagnosed by the treating physician. The exposure was sex (men versus women).

This study is reported according to the STROBE guidelines.

### National arthroplasty registers

Registries within the Network of Orthopaedic Registries of Europe (NORE) and affiliated registries were contacted to participate in this study. NORE is an international network for registries within EFORT (European Federation of National Associations of Orthopaedics and Traumatology), which focuses on medical device surveillance and outcomes of arthroplasties to improve patient care, education on arthroplasty outcome to different stakeholders (e.g., clinicians, regulatory bodies), and research [12].

National and regional arthroplasty registries prospectively collect data on arthroplasties, collectively have data on millions of patients, and have substantial long-term follow-up, in some cases exceeding 20 years. In addition to the type of implant and patient characteristics, they register outcomes including revision and reason for revision. National arthroplasty registries collect data on whether an implant has been revised or not, the reason for revision (e.g., infection, aseptic loosening, recurrent dislocation etc.) and patient demographic and implant-related variables. A map of countries that participate in NORE can be found here (https://efortnet.efort.org/nore-map/#/nore/map-all) and a list of most arthroplasty registries can be found here (https://nore.efort.org/arthroplasty-registries). For this study we contacted 19 eligible registries with available contact information.

Table 1 gives details of the participating registries.

**Table 1:** Demographics of registries

Register	Country/region	Start	Hip			Knee			Shoulder			Ankle		
			n	Mean age (SD)	% men	n	Mean age (SD)	% men	n	Mean age (SD)	% men	n	Mean age (SD)	% men
AOANJRR	Australia	1999	426,379	68.2 (10.7)	45.9	699,283	68.6 (9.1)	43.9	29,768	71.2 (9.2)	41.5	2,383	67.2 (9.0)	61.7
CJRR	Canada ^a^	2012	159,296	67.7 (15.2)	45.1	266,632	68.3 (12.7)	39.0	NA	NA	NA	NA	NA	NA
EPRD	Germany	2012	216,167	70.0 (10.1)	37.5	196,221	69.8 (9.3)	32.9	NA	NA	NA	NA	NA	NA
NAR	Norway	2005	97,011	69.8 (9.7)	34.5	75,004	68.8 (9.3)	39.8	3,729	70.2 (9.7)	39.3	321	65.2 (11.2)	59.8
NJR	U. K.	2003	1,142,363	68.4 (10.9)	40.7	1,310,663	69.0 (9.4)	43.8	27,343	71.4 (9.5)	30.4	6,307	68.3 (9.4)	63.2
NZJR	New Zealand	1999	127,276	67.0 (10.7)	48.0	112,465	68.0 (9.2)	48.9	5,816	70.8 (8.8)	38.8	NA	NA	NA
Orthopride	Belgium	2015	97,156	68.3 (10.9)	41.3	101,533	68.5 (9.5)	36.4	NA	NA	NA	NA	NA	NA
RACat	Catalonia ^b^	2005	57,944	68.5 (11.4)	40.2	65,564	72.0 (7.7)	29.2	NA	NA	NA	NA	NA	NA
RIAP	Italy ^c^	2010	14,181	69.1 (10.4)	48.8	5,744	71.0 (8.5)	39.3	NA	NA	NA	NA	NA	NA

AOANJRR = Australian Orthopaedic Association National Joint Replacement Registry, CJRR = Canadian Joint Replacement Registry, EPRD = Endoprothesenregister Deutschland, NAR = Norwegian Arthroplasty Register, NJR = National Joint Registry for England, Wales, Northern Ireland, the Isle of Man and Guernsey, NZJR = New Zealand Joint Registry, RACat = Catalan Arthroplasty Register, RIAP = Italian Arthroplasty Registry, n = number of arthroplasties, SD = standard deviation, NA = not available.

a For this study, Canada included replacements performed in 3 provinces with full coverage of CJRR prosthesis data (> 95%) during 2012 to 2019 data years (Ontario, Manitoba, British Columbia). This represents 74% of all replacements done in Canada.

b National Spanish registry is no longer up to date; RACat is a regional registry with more recent data.

c Data from the Provincial Government South Tyrol Arthroplasty Registry and the Registry of the Autonomous Province Trento.

### Data analyses

To comply with privacy regulations concerning data sharing, national arthroplasty registries performed the analyses locally (Cox regression analysis, proportional hazard assumption), according to their own legal framework, and reported the meta-data with an a priori designed data form to the leading institution (see Supplementary data) [13].

On a local registry level, the analyses consisted of a Cox regression to estimate unadjusted and adjusted hazard ratios (HR) for revision due to PJI for men compared with women. HRs and their standard errors were reported for each implant site (hip, knee, shoulder, ankle) and follow-up (1, 5, and 10 years) separately and adjusted for age, BMI, ASA-score, type of fixation, and type of implant.

Age is associated with hormone status, which may have an effect on the immune response and therefore on the association between sex and risk of revision due to infection [9]. Increased BMI is a known risk for infections, which may be different in men and women undergoing arthroplasty surgery for osteoarthritis [14]. The ASA score is associated with comorbidities of the patient, which may have an influence on the immune response and risk of PJI [15]. Type of fixation, cemented or uncemented, could be indirectly associated with sex (e.g., cemented is preferred in osteoporotic bone, which is more prevalent in females) and may be associated with the risk of infection [16]. The type of implant used may be sex-specific and may also be associated with different infection rates [15]. The type of implant was applicable to total hip and shoulder arthroplasty, but not to knee or ankle arthroplasty.

### Statistics

We used a random-effects model to pool the HR of individual registries for each arthroplasty and follow-up in order to estimate an overall HR along with its associated 95% confidence interval (CI) and, in case of heterogeneity, its 95% prediction interval (PI) [17,18]. According to recent recommendations no P values were reported [19]. The amount of statistical heterogeneity was assessed through inspection of forest plots and by calculating the I^2^ statistic, which estimates how much of the total variance in the effect size estimates is due to heterogeneity. We explored potential sources of heterogeneity when I^2^ was more than 40% through sub-group analyses and with random-effects meta-regression on predefined factors as reported by the national arthroplasty registries (e.g., completeness of the registry) according to the Cochrane Handbook. These analyses were performed with the metafor package in R statistics (R Foundation for Statistical Computing, Vienna, Austria) [17]. Details on data completeness for each registry can be found in their annual reports and was generally above 95% [20].

### Ethics, data sharing, funding, and disclosures

For this multinational arthroplasty registry study approval by the ethics committee was not required. All relevant data is in the manuscript or online Supplementary data. There was no external funding for this work. Hence, no sponsor took part in the design or conduct of the study; nor in the collection, management, analysis, or interpretation of the data; nor in the preparation, review, or approval of the manuscript. All authors had full access to all the data in the study and had final responsibility for the decision to submit for publication. All authors declared having received no support for this manuscript.

The following authors stated some form of funding: EL and AB are conducting a study investigating outcome of hip replacements according to the type of bearing materials used during primary hip arthroplasty, funded by Ceramtec GmBH. MD receives royalties or license from Stryker. EB received grants or contract from Zimmer, Smith & Nephew, DePuy, and Hip Innovation Technology, and consulting fees from Stryker Canada. BP received grants or contracts from ZonMW. AB received grants or contract from the National Institute for Health Research and the National Joint Registry. JV received grants or contract from Corin, Smith & Nephew, and Aqtor.None of the fundings mentioned above had any influence on the conduct of this study. Complete disclosure of interest forms according to ICMJE are available on the article page, doi: 10.2340/17453674.2024.42183

## Results

**Figure 1:**
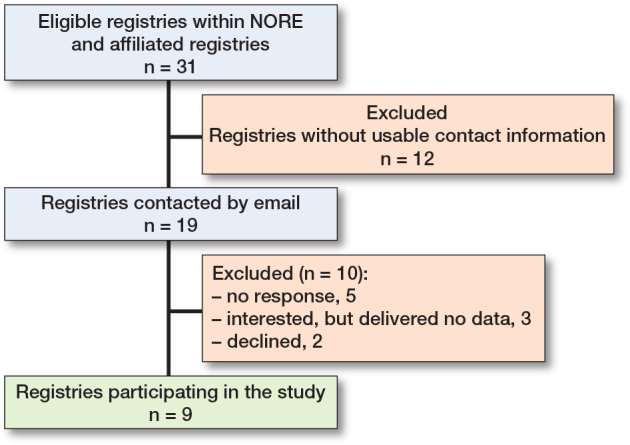
Flowchart registry inclusions.

9 registries from Australia, Belgium, Canada, Catalonia (Spain), Germany, Italy, New Zealand, Norway, and the United Kingdom collectively included 2,134,313 primary hip arthroplasties, 2,658,237 primary knee arthroplasties, 57,889 primary shoulder arthroplasties, and 8,445 primary ankle arthroplasties (Figure 1). All implants were registered between 1999 and 2021.

### Hip arthroplasty

**Figure 2:**
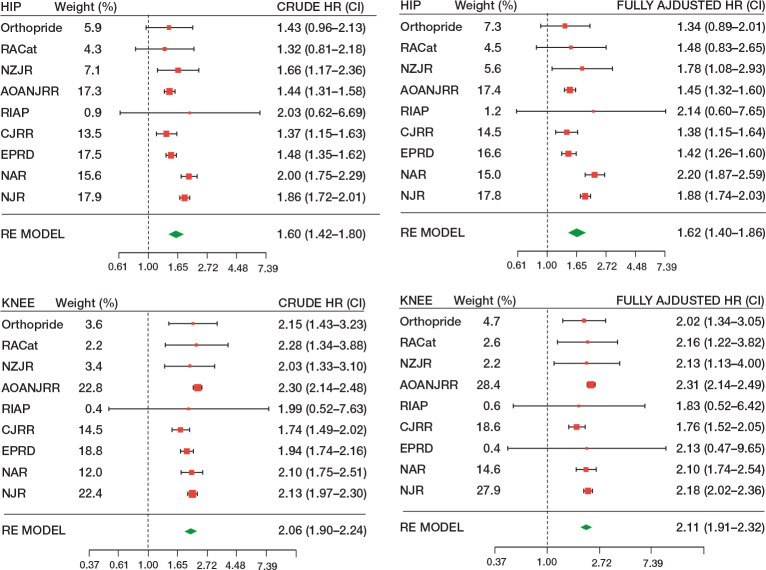
Forest plots: pooled unadjusted estimates and adjusted estimates for hip and knee arthroplasties at 1-year follow-up. HR > 1, equals a higher risk for men compared with women. Weights of registries in %. For abbreviations, see Table 1.

In the unadjusted model at 1-year follow-up, men had a 60% higher risk of revision for infection compared with women based on 2,134,313 hip arthroplasties from 9 registries (HR 1.60, CI 1.42–1.80), and in the fully adjusted model HR is 1.62 (CI 1.40–1.86) (Figure 2 and Table 2). The risk of revision for infection remained higher for men compared with women at 5-year and 10-year follow-up (5-year HR 1.63, CI 1.46–1.81; 10-year HR 1.84, CI 1.66–2.05, Table 2). The risk of revision for infection also remained higher for men compared with women when adjusted separately for age, BMI, ASA score, type of fixation, and type of implant (Table 3).

**Table 2:** Pooled unadjusted hazard ratios (HR) per follow-up time

Joint	Registries	Prostheses	Pooled HR (CI)	I^2^ (%)	95% PI
Follow-up	n	n			
Hip					
1 year	9	2,134,313	1.60 (1.42–1.80)	77	1.20–2.13
5 years	7	1,111,954	1.63 (1.46–1.81)	77	1.26–2.10
10 years	5	426,237	1.84 (1.66–2.05)	54	1.52–2.24
Knee					
1 year	9	2,658,237	2.06 (1.90–2.46)	54	1.73–2.46
5 years	7	1,378,891	1.84 (1.77–1.91)	0.0	
10 years	5	507,569	1.83 (1.70–1.96)	30	
Shoulder					
1 year	3	57,889	4.51 (2.99–6.80)	0.0	
5 years	4	28,864	4.13 (2.99–5.71)	0.0	
10 years	3	9,065	3.03 (1.77–5.19)	0.0	
Ankle					
1 year	3	8,445	0.87 (0.46–1.62)	0.0	
5 years	3	3,817	0.84 (0.55–1.29)	0.0	
10 years	2	279	0.82 (0.53–1.26)	0.0	

CI = 95% confidence interval; I^2^ = heterogeneity; PI = prediction interval.

**Table 3:** Pooled hazard ratios (HR) at 1-year follow-up

Joint	Registries	Prostheses	Pooled HR (CI)	I^2^ (%)
Adjustments	n	n		
Hip				
Unadjusted	9	2,134,313	1.60 (1.42–1.80)	77
Adjusted for ^a^				
BMI	3	1,361,889	1.58 (1.24–2.01)	88
Age	9	2,134,313	1.61 (1.42–1.83)	81
ASA	3	1,277,874	1.91 (1.79–2.04)	0.0
Type of fixation	9	2,134,313	1.63 (1.43–1.85)	80
Type of implant	5	1,701,123	1.69 (1.43–2.00)	84
Fully adjusted	9	2,134.,313	1.62 (1.40–1.86)	81
Knee				
Unadjusted	9	2,658,237	2.06 (1.90–2.46)	54
Adjusted for ^a^				
BMI	3	1,503,858	2.09 (1.94–2.26)	5.2
Age	9	2,658,237	2.07 (1.92–2.24)	49
ASA	3	1,413,726	2.13 (1.98–2.29)	0.0
Type of fixation	9	2,658,237	2.07 (1.90–2.25)	57
Fully adjusted	9	2,658,237	2.11 (1.91–2.32)	49
Shoulder				
Unadjusted	3	57,889	4.51 (2.99–6.80)	0.0
Adjusted for ^a^				
BMI	1	24,945	5.86 (2.57–13.4) ^b^	
Age	3	57,889	4.28 (2.82–6.49)	0.0
ASA	2	28,201	5.75 (2.79–11.9)	0.0
Type of fixation	2	54,633	4.33 (2.83–6.63)	0.0
Type of implant	3	57,889	4.90 (3.25–7.39)	0.0
Fully adjusted	3	57,889	4.25 (2.81–6.45)	0.0
Ankle				
Unadjusted	3	8,445	0.87 (0.46–1.62)	0.0
Adjusted for ^a^				
BMI	1	5,785	0.81 (0.32–2.04) ^b^	
Age	2	8,149	0.94 (0.49–1.79)	0.0
ASA	1	5,785	0.80 (0.32–1.97) ^b^	
Type of fixation	2	8,149	0.93 (0.48–1.77)	0.0
Fully adjusted	2	8,149	0.92 (0.48–1.80)	0.0

CI = 95% confidence interval; I^2^ = heterogeneity

a Hazard ratio for sex adjusted for BMI, age, ASA, implant fixation, and type of implant.

b Results based on only a single registry, i.e., the reported HR is not pooled across all registries.

### Knee arthroplasty

In the unadjusted model at 1-year follow-up, men had a 106% higher risk of revision for infection compared with women based on 2,658.237 knee arthroplasties from 9 registries (HR 2.06, CI 1.90–2.46), and in the fully adjusted model HR is 2.11 (CI 1.91–2.32) (Figure 2 and Table 2). The risk of revision for infection remained higher for men compared with women at 5-year and 10-year follow-up (5-year HR 1.84, CI 1.77–1.91; 10-year HR 1.83, CI 1.70–1.96; Table 2). The risk of revision for infection remained higher for men compared with women when adjusted separately for age, BMI, ASA score, and type of fixation (Table 3).

### Shoulder arthroplasties

In the unadjusted model at 1-year follow-up men had a 351% higher risk of revision for infection compared with women based on 57,889 shoulder arthroplasties from 3 registries (HR 4.51, CI 2.99–6.80), and in the fully adjusted model HR is 4.25 (CI 2.81–6.45) (Table 2). The risk of revision for infection remained higher for men compared with women at 5-year and 10-year follow-up (5-year HR 4.13, CI 2.99–5.71; 10-year HR 3.03, CI 1.77–5.19; Table 2). In addition, the risk of revision for infection remained higher for men compared with women when adjusted for age, ASA score, type of fixation, and type of implant (Table 3).

### Ankle arthroplasties

At 1-, 5-, and 10-year follow-up there was no relevant difference between men and women for risk of revision for infection based on 8,445 ankle arthroplasties from 3 registries (unadjusted model 1-year HR 0.87, CI 0.46–1.62; 5-year HR 0.84, CI 0.55–1.29; 10-year HR 0.82, CI 0.53–1.26; fully adjusted model 1-year HR 0.92, CI 0.48–1.80; Table 2).

### Sensitivity analyses

The meta-regression and sub-group analyses could not identify any effect modifiers: for both total hip replacement and total knee replacements heterogeneity could not be explained by difference in mean age, percentage of men, registry completeness, or geographical location (Europe vs non-Europe).

## Discussion

We aimed to determine sex differences for periprosthetic joint infections after primary arthroplasty of the hip, knee, ankle, and shoulder in osteoarthritis patients in an international perspective. We showed that there was a higher risk, both crude and adjusted, for revision due to infection in men compared with women after primary hip, knee, and shoulder arthroplasty. For total ankle arthroplasty we did not find sex-based differences. When followed over time, up to 10-year follow-up, the risk of developing a PJI demanding revision remained higher for men.

Our findings concur with the published findings in knee arthroplasty from the Finnish Arthroplasty Registry (FAR), which found a higher risk for reoperation because of infection after primary (HR 1.54, CI 1.21–1.98) and revision (HR 2.23, CI 1.30–3.62) total knee replacement and with a cohort of 56,216 total knee replacements from the USA (HR 1.89, CI 1.54–2.32) [21,22]. Our findings are also consistent with previously published data from the National Joint Registry of England and Wales (HR of 1.8, CI 1.7–2.0), as this registry contributed the majority of cases presented in our analysis [23].

Similarly, our findings concur with the published registry findings on hip replacements, where FAR reported a higher risk of revision due to infection for men with an HR of 1.7 (CI 1.4–2.0); a cohort of 132,826 patients from New York and California (USA) reported an HR of 1.3 (CI 1.1–1.5) [24]. The National Joint Registry of England and Wales reported an HR of 1.7 (CI 1.6–1.8) and the Danish Hip Arthroplasty Registry an HR of 1.5 (CI 1.3–1.8) [25,26].

Data on infection following primary shoulder arthroplasty is sparse. A study from California of 3,906 patients reported a higher risk for infection for men compared with women (HR 2.59, CI 1.27–5.31). A single-center series from the USA of 2,207 total shoulder arthroplasties also reported a higher risk of PJI for men compared with women (HR 2.67, CI 1.22–5.87) [27,28]. However, the differential risk in our multi-register study was far higher: HR 4.22 (CI 2.63–6.78).

Regarding total ankle arthroplasty our results did not show any sex difference, but confidence intervals were large, suggesting uncertainty in the findings.

### Potential mechanisms and relevance

As to the potential mechanism behind sex difference we postulate that this is due to difference in the immune system between men and women. Nutrition and composition of the microbiome influence the development of the immune system as well. Differences in immune response partially alter during life, influenced by age and hormonal status, especially during the menopause. Hormone replacement therapy has been shown to reverse menopause-related immunological changes, causing beneficial effects on the immune system [9]. These alterations may have an effect on the risk difference for PJI and should be targeted in future research.

Compared with the other anatomical regions, the sex difference in risk of revision for PJI was most pronounced in total shoulder arthroplasty (HR 4.51). It may be that for certain micro-organisms, such as *Cutibacterium acnes*, the sex difference is amplified due to, e.g., a hormonal component, which could explain the large sex difference for total shoulder arthroplasty [27]. Indeed, it has been shown that the bacterial load and intraoperative growth of *Cutibacterium acnes* is higher for men compared with women, possibly giving rise to an increased risk of PJI for men [29].

### Strengths

This is the largest and most comprehensive evaluation of sex-based differences in risk of revision for PJI after primary total joint replacement including more than 4.8 million patients from 9 international registries giving enough statistical power to risk of revision due to PJI. Other strengths include the long-term follow-up of 10 years, as well as the advanced methodology we used, allowing the combining of data from several national joint registries with pre-specified analyses. In contrast to previous studies, we have adjusted for confounders both separately and combined; the latter did not change the results of our findings. Although other studies have already implicated the male sex as a risk factor for PJI, this multinational study confirms this sex-based risk difference on a far larger scale. The latter allows for a more optimal correction for several confounders as well as evaluating the striking risk difference between different anatomical regions.

### Limitations

First, because this study is observational, no causative relationship can be established. Second, not all possible confounders are registered in all the registries, therefore the fully adjusted sex HR differs from one registry to another, and the possibility of residual confounding exists. For instance, we were unable to control for diabetes, smoking, alcohol consumption, socioeconomic factors, and occupational hazards. Third, we used ASA score to adjust for comorbidities, because this variable is available in most registries. However, ASA score is a crude method to account for comorbidities and lacks details. Fourth, revision for PJI or suspicion of PJI reflects a clinical diagnosis sufficient for the surgeon to perform revision surgery. A preoperative, pre-surgical (differential) diagnosis of PJI cannot be corrected or verified postoperatively, when the actual microbiological cultures are available. This may lead to an imprecise estimation of the incidence of PJI, or even a misclassification. Fifth, the magnitude of the pooled hazard ratio varied according to the joint considered, with the greatest differential in shoulders with over 4-fold difference, followed by knees, over double, and hips 1.6 times. In contrast no sex difference could be identified in ankle replacements.

### Conclusion

Men have a higher risk of revision due to infection than women after primary hip, knee, and shoulder arthroplasty. No evidence of difference was found for ankle arthroplasty. These elevated relative risks persist in the fully adjusted investigations and over the 10-year postoperative period studied.

*In perspective,* based on the results of our study, future studies evaluating outcome of PJI should adjust for sex or present their outcomes separately for each sex. Furthermore, evaluation should be made for each anatomical region, given the variance in risk difference. While sex is a non-modifiable risk factor for PJI, it is still important for clinicians to be aware of the higher PJI risk for men, especially for shoulder arthroplasty. Awareness of all risk factors, including sex, would allow for a patient-tailored approach in optimizing the risk of PJI preoperatively. For instance, in male patients with several other risk factors, it is particularly important to reduce modifiable risk factors such as obesity, smoking, or proton pump inhibitor use [30].

### Supplementary data

Table with all hazard ratios provided by the registries (Appendix 1) and survey sent out to all the participating registries (Appendix 2) are available as Supplementary data on the article page, doi: 10.2340/17453674.2024.42183

## Supplementary Material





## Notes

BP and RN acted as principal investigators; they take responsibility for the integrity of the data and the accuracy of the data analysis, are responsible for the study concept and design, and supervised the study. All authors contributed to the acquisition, analysis, or interpretation of the data, and critical revision of the manuscript for important intellectual content. AR and BP drafted the manuscript, and all authors reviewed it. All authors also provided administrative, technical, and material support. BP attests that all listed authors meet authorship criteria and that no others meeting the criteria have been omitted.

The authors thank Michael Terner from the Canadian Joint Replacement Registry (Canadian Institute for Health Information) for help with the data analyses and helpful suggestions for the manuscript. The authors also thank Roberto Picus and Cristiana Armaroli who provided the Italian Arthroplasty Registry (RIAP) with the data requested for the study from the Provincial Government South Tyrol Arthroplasty Registry and the Registry of the Autonomous Province Trento, respectively.

Handling co-editors: Keijo Mäkelä and Philippe Wagner

*Acta* thanks Srinivas B S Kambhampati and Svend Erik Østgaard for help with peer review of this manuscript.
