# Supplementary material for: Sex-based differences in risk of revision for infection after hip, knee, shoulder, and ankle arthroplasty in osteoarthritis patients: a multinational registry study of 4,800,000 implants
Source: Acta Orthop. 2024 Dec 10;95:730–6. doi: 10.2340/17453674.2024.42183 (PMC11632196; doi:10.2340/17453674.2024.42183)
Supplement: Supplementary file 1 [file ActaO-95-42183-s1.pdf]

Appendix 1. Unadjusted and adjusted hazard ratios and corresponding standard errors associated with sex (male vs female (reference)) by registries and joint sites

HIP	Unadjusted	BMI	Age	ASA	Type of fixation	Type of implant	Fully adjusted
**AOANJRR**							
1 year	1.44 (0.048)	NA	1.42 (0.048)	NA	1.48 (0.048)	1.44 (0.048)	1.45 (0.048)
5 years	1.57 (0.070)	NA	1.55 (0.070)	NA	1.62 (0.070)	1.57 (0.070)	1.59 (0.071)
10 years	1.91 (0.114)	NA	1.88 (0.114)	NA	1.96 (0.114)	1.91 (0.114)	1.92 (0.114)
**CJRR**							
1 year	1.37 (0.090)	NA	1.36 (0.090)	NA	1.38 (0.090)	NA	1.38 (0.090)
5 years	1.40 (0.081)	NA	1.38 (0.082)	NA	1.42 (0.082)	NA	1.40 (0.082)
10 years	NA	NA	NA	NA	NA	NA	NA
**EPRD**							
1 year	1.48 (0.046)	1.31 (0.060)	1.54 (0.046)	NA	1.49 (0.046)	NA	1.42 (0.061)
5 years	1.50 (0.043)	1.33 (0.058)	1.54 (0.043)	NA	1.52 (0.043)	NA	1.44 (0.059)
10 years	NA	NA	NA	NA	NA	NA	NA
**NAR**							
1 year	2.0 (0.068)	NA	2.1 (0.068)	2.0 (0.068)	2.2 (0.083)	2.0 (0.084)	2.2 (0.084)
5 years	2.0 (0.061)	NA	2.1 (0.061)	2.0 (0.061)	2.3 (0.074)	2.0 (0.061)	2.3 (0.075)
10 years	2.1 (0.059)	NA	2.2 (0.060)	2.1 (0.060)	2.4 (0.072)	2.1 (0.059)	2.4 (0.072)
**NJR**							
1 year	1.86 (0.04)	1.81 (0.04)	1.87 (0.04)	1.89 (0.04)	1.85 (0.04)	1.92 (0.04)	1.88 (0.04)
5 years	1.79 (0.03)	1.79 (0.03)	1.76 (0.03)	1.81 (0.03)	1.80 (0.03)	1.82 (0.03)	1.78 (0.03)
10 years	1.78 (0.03)	1.76 (0.03)	1.79 (0.03)	1.79 (0.03)	1.79 (0.03)	1.79 (0.03)	1.75 (0.03)
**NZJR**							
1 year	1.66 (0.179)	1.75 (0.247)	1.68 (0.183)	1.66 (0.188)	1.62 (0.176)	NA	1.78 (0.255)
5 years	1.67 (0.140)	1.95 (0.245)	1.64 (0.139)	1.71 (0.158)	1.66 (0.140)	NA	1.93 (0.245)
10 years	1.74 (0.132)	2.00 (0.244)	1.70 (0.130)	1.74 (0.150)	1.73 (0.132)	NA	1.99 (0.246)
**Orthopride**							
1 year	1.43 (0.204)	NA	1.34 (0.207)	NA	1.41 (0.204)	1.45 (0.205)	1.34 (0.208)
5 years	NA	NA	NA	NA	NA	NA	NA
10 years	NA	NA	NA	NA	NA	NA	NA
**RACat**							

1 year	1.33 (0.254)	NA	1.37 (0.270)	NA	1.45 (0.284)	1.35 (0.260)	1.48 (0.295)
5 years	1.24 (0.202)	NA	1.28 (0.214)	NA	1.37 (0.227)	1.26 (0.206)	1.39 (0.235)
10 years	1.28 (0.203)	NA	1.30 (0.212)	NA	1.39 (0.224)	1.30 (0.207)	1.40 (0.231)
**RIAP**							
1 year	2.03 (0.607)	NA	2.12 (0.644)	NA	2.04 (0.608)	NA	2.14 (0.650)
5 years	2.02 (0.604)	NA	2.09 (0.637)	NA	2.05 (0.612)	NA	2.14 (0.648)
10 years	NA	NA	NA	NA	NA	NA	NA

KNEE	Unadjusted	BMI	Age	ASA	Type of fixation	Fully adjusted
**AOANJRR**						
1 year	2.30 (0.038)	NA	2.28 (0.038)	NA	2.34 (0.038)	2.31 (0.038
5 years	1.88 (0.040)	NA	1.85 (0.040)	NA	1.91 (0.040)	1.88 (0.040)
10 years	1.71 (0.074)	NA	1.68 (0.074)	NA	1.74 (0.074)	1.71 (0.074)
**CJRR**						
1 year	1.74 (0.077)	NA	1.75 (0.077)	NA	1.75 (0.077)	1.76 (0.077)
5 years	1.84 (0.061)	NA	1.86 (0.061)	NA	1.84 (0.061)	1.86 (0.061)
10 years	NA	NA	NA	NA	NA	NA
**EPRD**						
1 year	1.94 (0.056)	2.05 (0.077)	1.95 (0.056)	NA	1.93(0.056)	2.13 (0.077)
5 years	1.82 (0.047)	2.00 (0.071)	1.83 (0.047)	NA	1.82 (0.048)	2.06 (0.071)
10 years	NA	NA	NA	NA	NA	NA
**NAR**						
1 year	2.1 (0.092)	NA	2.2 (0.092)	2.1 (0.096)	2.1 (0.096)	2.1 (0.097)
5 years	1.9 (0.076)	NA	2.0 (0.076)	2.0 (0.076)	1.9 (0.080)	1.9 (0.081)
10 years	2.1 (0.074)	NA	2.1 (0.074)	2.1 (0.074)	2.1 (0.078)	2.0 (0.078)
**NJR**						
1 year	2.13 (0.04)	2.17 (0.04)	2.14 (0.04)	2.14 (0.04)	2.14 (0.04)	2.18 (0.04)
5 years	1.81 (0.03)	1.83 (0.03)	1.79 (0.03)	1.81 (0.02)	1.82 (0.03)	1.84 (0.03)
10 years	1.79 (0.02)	1.81 (0.02)	1.78 (0.02)	1.79 (0.02)	1.80 (0.02)	1.81 (0.02)
**NZJR**						

1 year	2.03 (0.216)	2.18 (0.328)	2.02 (0.215)	1.97 (0.22)	2.03 (0.216)	2.13 (0.322)
5 years	1.83 (0.134)	2.04 (0.235)	1.82 (0.133)	1.81 (0.143)	1.84 (0.134)	2.00 (0.232)
10 years	1.77 (0.119)	1.96 (0.218)	1.75 (0.118)	1.74 (0.129)	1.77 (0.119)	1.92 (0.216)
**Orthopride**						
1 year	2.15 (0.208)	NA	20.4 (0.209)	NA	2.12 (0.209)	2.02 (0.210)
5 years	NA	NA	NA	NA	NA	NA
10 years	NA	NA	NA	NA	NA	NA
**RACat**						
1 year	2.28 (0.272)	NA	2.26 (0.270)	NA	2.16 (0.291)	2.14 (0.290)
5 years	1.92 (0.190)	NA	1.90 (0.187)	NA	1.96 (0.221)	1.94 (0.218)
10 years	1.90 (0.184)	NA	1.87 (0.181)	NA	1.91 (0.210)	1.88 (0.207)
**RIAP**						
1 year	1.99 (0.686)	NA	1.83 (0.636)	NA	1.99 (0.690)	1.83 (0.640)
5 years	NA	NA	NA	NA	NA	NA
10 years	NA	NA	NA	NA	NA	NA

**SHOULDER**	**Unadjusted**	**BMI**	**Age**	**ASA**	**Type of fixation**	**Type of implant**	**Fully adjusted**
**AOANJRR**							
1 year	4.00 (0.254)	NA	3.71 (0.256)	NA	3.99 (0.254)	4.26 (0.254)	3.70 (0.256)
5 years	3.95 (0.249)	NA	3.654 (0.252)	NA	3.95 (0.249)	4.24 (0.249)	3.69 (0.251)
10 years	3.01 (0.369)	NA	2.76 (0.371)	NA	3.00 (0.369)	3.28 (0.370)	2.83 (0.371)
**CJRR**							
1 year	NA	NA	NA	NA	NA	NA	NA
5 years	NA	NA	NA	NA	NA	NA	NA
10 years	NA	NA	NA	NA	NA	NA	NA
**EPRD**							
1 year	NA	NA	NA	NA	NA	NA	NA
5 years	NA	NA	NA	NA	NA	NA	NA
10 years	NA	NA	NA	NA	NA	NA	NA
**NAR**							
1 year	7.1 (0.780)	NA	5.9 (0.810)	7.1 (0.780)	NA	8.6 (0.789)	6.7 (0.808)
5 years	3.4 (0.494)	NA	2.4 (0.518)	3.4 (0.494)	2.9 (0.558)	4.0 (0.502)	2.7 (0.519)
10 years	3.2 (0.433)	NA	2.3 (0.454)	3.2 (0.434)	3.0 (0.436)	3.8 (0.441)	2.5 (0.455)
**NJR**							

1 year	5.49 (0.42)	5.33 (0.42)	5.86 (0.43)	5.41 (0.42)	5.39 (0.42)	6.13 (0.42)	5.52 (0.43)
5 years	4.61 (0.25)	4.54 (0.29)	4.23 (0.26)	4.66 (0.25)	4.55 (0.25)	5.02 (0.25)	4.16 (0.26)
10 years	4.38 (0.23)	4.32 (0.23)	3.99 (0.24)	4.37 (0.24)	4.31 (0.23)	4.75 (0.23)	3.90 (0.24)
**NZJR**							
1 year	NA	NA	NA	NA	NA	NA	NA
5 years	2.46 (1.537)	NA	2.17 (1.399)	NA	NA	2.53 (1.584)	2.11 (1.354)
10 years	2.11 (1.245)	NA	1.80 (1.096)	NA	NA	2.17 (1.280)	1.76 (1.065)
**Orthopride**							
1 year	NA	NA	NA	NA	NA	NA	NA
5 years	NA	NA	NA	NA	NA	NA	NA
10 years	NA	NA	NA	NA	NA	NA	NA
**RACat**							
1 year	NA	NA	NA	NA	NA	NA	NA
5 years	NA	NA	NA	NA	NA	NA	NA
10 years	NA	NA	NA	NA	NA	NA	NA
**RIAP**							
1 year	NA	NA	NA	NA	NA	NA	NA
5 years	NA	NA	NA	NA	NA	NA	NA
10 years	NA	NA	NA	NA	NA	NA	NA

**ANKLE**	**Unadjusted**	**BMI**	**Age**	**ASA**	**Type of fixation**	**Fully adjusted**
**AOANJRR**						
1 year	1.07 (0.476)	NA	1.10 (0.476)	NA	1.07 (0.476)	1.10 (0.476)
5 years	0.96 (0.913)	NA	0.98 (0.913)	NA	0.96 (0.913)	0.98 (0.913)
10 years	NA	NA	NA	NA	NA	NA
**CJRR**						
1 year	NA	NA	NA	NA	NA	NA
5 years	NA	NA	NA	NA	NA	NA
10 years	NA	NA	NA	NA	NA	NA
**EPRD**						
1 year	NA	NA	NA	NA	NA	NA
5 years	NA	NA	NA	NA	NA	NA
10 years	NA	NA	NA	NA	NA	NA
**NAR**						
1 year	0.38 (1.225)	NA	NA	NA	NA	NA
5 years	0.34 (1.225)	NA	NA	NA	NA	NA
10 years	NA	NA	NA	NA	NA	NA
**NJR**						
1 year	0.80 (0.46)	0.75 (0.47)	0.81 (0.46)	0.80 (0.46)	0.81 (0.46)	0.77 (0.47)

5 years	0.86 (0.23)	0.86 (0.23)	0.88 (0.23)	0.86 (0.23)	0.86 (0.23)	0.88 (0.23)
10 years	0.82 (0.22)	0.81 (0.22)	0.84 (0.22)	0.81 (0.22)	0.82 (0.22)	0.83 (0.22)
**NZJR**						
1 year	NA	NA	NA	NA	NA	NA
5 years	NA	NA	NA	NA	NA	NA
10 years	NA	NA	NA	NA	NA	NA
**Orthopride**						
1 year	NA	NA	NA	NA	NA	NA
5 years	NA	NA	NA	NA	NA	NA
10 years	NA	NA	NA	NA	NA	NA
**RACat**						
1 year	NA	NA	NA	NA	NA	NA
5 years	NA	NA	NA	NA	NA	NA
10 years	NA	NA	NA	NA	NA	NA
**RIAP**						
1 year	NA	NA	NA	NA	NA	NA
5 years	NA	NA	NA	NA	NA	NA
10 years	NA	NA	NA	NA	NA	NA

(AOANJRR = Australian Orthopaedic Association National Joint Replacement Registry, CJRR = Canadian Joint Replacement Registry, EPRD = Endoprothesenregister Deutschland, NAR = Norwegian Arthroplasty Register, NJR = National Joint Registry for England, Wales, Northern Ireland, the Isle of Man and Guernsey, NZJR = New Zealand Joint Registry, RACat = Catalan Arthroplasty Register, RIAP = Italian Arthroplasty Registry, NA = not available)
