# Supplementary material for: Sex-based differences in risk of revision for infection after hip, knee, shoulder, and ankle arthroplasty in osteoarthritis patients: a multinational registry study of 4,800,000 implants
Source: Acta Orthop. 2024 Dec 10;95:730–6. doi: 10.2340/17453674.2024.42183 (PMC11632196; doi:10.2340/17453674.2024.42183)
Supplement: Supplementary file 2 [file ActaO-95-42183-s2.pdf]

Survey risk of revision due to infection in men and women

Thank you for helping with this research to examine whether the risk of revision due to infection is higher for men than women. We will focus on revisions of primary arthroplasties placed for osteoarthritis. The survey we ask you to fill in, consists of a few general questions and 4 parts of specific questions about groups of prostheses. For each group broadly similar questions are asked, they will only need to be answered if the data is available. The question will specify what data is required and how it should be provided.

		**Question**	**Answer**
	1	Please provide the full name of your registry.	
	2	Please provide the URL of your registry website.	
	3	Can you give any information about the completeness of your registry? For instance, an article or percentages.	

	4	Have you recorded details of revision rates due to infection in primary hip arthroplasty for osteoarthritis?		If not, proceed to question 5<br>If yes, proceed to question 4.1
	4.1	What is the mean age of all primary hip arthroplasties for osteoarthritis in your registry?<br>*(provide mean age and standard deviation)*		
	4.2	What is the gender distribution of all primary hip arthroplasties for osteoarthritis in your registry?<br>*(provide in numbers with percentage)*		
	4.3	What is the start of data collection?		
	4.4	Do you have data on revision rate due to infection of primary hip arthroplasties for osteoarthritis at 1 year?		If not, proceed to question 4.5<br>If yes, proceed to question 4.4.1
		4.4.1	What is the hazard ratio of revision due to infection for men compared to women?<br>*(Provide a hazard ratio with standard error. Also provide #men and #women of which the follow-up of 1 year is complete.)*	
		4.4.2	What is the hazard ratio of revision due to infection for men compared to women, adjusted for:<br>- BMI<br>- Age<br>- ASA score (I, II, III, IV)<br>- Type of fixation (cemented or non-cemented)	*BMI*
				Age
				ASA score

		- Type of prosthesis (hemiarthroplasty, total hip arthroplasty or resurfacing arthroplasty)<br>- The above combined (add which you've included)<br><br>*(Provide a hazard ratio with standard error)*	*Type of fixation*<br>*Type of prosthesis*<br>*Combined*
4.5	Do you have data on revision rate due to infection of primary hip arthroplasties for osteoarthritis at 5 years?		If not, proceed to question 4.6<br>If yes, proceed to question 4.5.1
	4.5.1	What is the risk of revision due to infection for men compared to women?<br>*(Provide a hazard ratio with standard error. Also provide #men and #women of which the follow-up of 5 years is complete.)*	
	4.5.2	What is the hazard ratio of revision due to infection for men compared to women, adjusted for:<br>- BMI<br>- Age<br>- ASA score (I, II, III, IV)<br>- Type of fixation (cemented or non-cemented)<br>- Type of prosthesis (hemiarthroplasty, total hip arthroplasty or resurfacing arthroplasty)<br>- The above combined (add which you've included)<br><br>*(Provide a hazard ratio with standard error)*	*BMI*<br>*Age*<br>*ASA score*<br>*Type of fixation*<br>*Type of prosthesis*<br>*Combined*
4.6	Do you have data on revision rate due to infection of primary hip arthroplasties for osteoarthritis at 10 years?		If not, proceed to question 5<br>If yes, proceed to question 4.6.1
	4.6.1	What is the risk of revision due to infection for men compared to women?<br>*(Provide a hazard ratio with standard error. Also provide #men and #women of which the follow-up of 10 years is complete.)*	

		4.6.2	What is the hazard ratio of revision due to infection for men compared to women, adjusted for:<br>- BMI<br>- Age<br>- ASA score (I, II, III, IV)<br>- Type of fixation (cemented or non-cemented)<br>- Type of prosthesis (hemiarthroplasty, total hip arthroplasty or resurfacing arthroplasty)<br>- The above combined (add which you've included)<br><br>*(Provide a hazard ratio with standard error)*	*BMI*<br>*Age*<br>*ASA score*<br>*Type of fixation*<br>*Type of prosthesis*<br>*Combined*

	5	Have you recorded details of revision rates due to infection in primary knee arthroplasty for osteoarthritis?		If not, proceed to question 6<br>If yes, proceed to question 5.1
	5.1	What is the mean age of all primary knee arthroplasties for osteoarthritis in your registry?<br>*(provide mean age and standard deviation)*		
	5.2	What is the gender distribution of all primary knee arthroplasties for osteoarthritis in your registry?<br>*(provide in numbers with percentage)*		
	5.3	What is the start of data collection?		
	5.4	Do you have data on revision rate due to infection of primary knee arthroplasties for osteoarthritis at 1 year?		If not, proceed to question 5.5<br>If yes, proceed to question 5.4.1
		5.4.1	What is the risk of revision due to infection for men compared to women?<br>*(Provide a hazard ratio with standard error. Also provide #men and #women of which the follow-up of 1 year is complete.)*	
		5.4.2	What is the hazard ratio of revision due to infection for men compared to women, adjusted for:<br>- BMI<br>- Age<br>- ASA score (I, II, III, IV)	*BMI*<br>*Age*

			- Type of fixation (cemented or non-cemented)<br>- Type of prosthesis (Total arthroplasty or hemiarthroplasty)<br>- The above combined (add which you've included)<br><br>*(Provide a hazard ratio with standard error)*	*ASA score*
				Type of fixation
				Type of prosthesis
				Combined
	5.5	Do you have data on revision rate due to infection of primary knee arthroplasties for osteoarthritis at 5 years?		If not, proceed to question 5.6<br>If yes, proceed to question 5.5.1
		5.5.1	What is the risk of revision due to infection for men compared to women?<br>*(Provide a hazard ratio with standard error. Also provide #men and #women of which the follow-up of 5 years is complete.)*	
		5.5.2	What is the hazard ratio of revision due to infection for men compared to women, adjusted for:<br>- BMI<br>- Age<br>- ASA score (I, II, III, IV)<br>- Type of fixation (cemented or non-cemented)<br>- Type of prosthesis (Total arthroplasty or hemiarthroplasty)<br>- The above combined (add which you've included)<br><br>*(Provide a hazard ratio with standard error)*	*BMI*
				Age
				ASA score
				Type of fixation
				Type of prosthesis
				Combined
	5.6	Do you have data on revision rate due to infection of primary knee arthroplasties for osteoarthritis at 10 years?		If not, proceed to question 6<br>If yes, proceed to question 5.6.1
		5.6.1	What is the risk of revision due to infection for men compared to women?<br>*(Provide a hazard ratio with standard error. Also provide #men and*	

		#women of which the follow-up of 10 years is complete.)	
	5.6.2	What is the hazard ratio of revision due to infection for men compared to women, adjusted for:<br>- BMI<br>- Age<br>- ASA score (I, II, III, IV)<br>- Type of fixation (cemented or non-cemented)<br>- Type of prosthesis (Total arthroplasty or hemiarthroplasty)<br>- The above combined (add which you've included)<br><br>*(Provide a hazard ratio with standard error)*	*BMI*<br><br>*Age*<br><br>*ASA score*<br><br>*Type of fixation*<br><br>*Type of prosthesis*<br><br>*Combined*

6	Have you recorded details of revision rates due to infection in primary shoulder arthroplasty for osteoarthritis?		If not, proceed to question 7<br>If yes, proceed to question 6.1
6.1	What is the mean age of all primary shoulder arthroplasties for osteoarthritis in your registry?<br>*(provide mean age and standard deviation)*		
6.2	What is the gender distribution of all primary shoulder arthroplasties for osteoarthritis in your registry?<br>*(provide in numbers with percentage)*		
6.3	What is the start of data collection?		
6.4	Do you have data on revision rate due to infection of primary shoulder arthroplasties for osteoarthritis at 1 year?		If not, proceed to question 6.5<br>If yes, proceed to question 6.4.1
	6.4.1	What is the risk of revision due to infection for men compared to women?<br>*(Provide a hazard ratio with standard error. Also provide #men and #women of which the follow-up of 1 year is complete.)*	
	6.4.2	What is the hazard ratio of revision due to infection for men compared to women, adjusted for:<br>- BMI<br>- Age<br>- ASA score (I, II, III, IV)	*BMI*<br><br>*Age*

			- Type of fixation (cemented or non-cemented)<br>- Type of prosthesis (Reversed arthroplasty, total anatomical arthroplasty or hemiarthroplasty)<br>- The above combined (add which you've included)<br><br>*(Provide a hazard ratio with standard error)*	*ASA score*
				Type of fixation
				Type of prosthesis
				Combined
	6.5	Do you have data on revision rate due to infection of primary shoulder arthroplasties for osteoarthritis at 5 years?		If not, proceed to question 6.6<br>If yes, proceed to question 6.5.1
		6.5.1	What is the risk of revision due to infection for men compared to women?<br>*(Provide a hazard ratio with standard error. Also provide #men and #women of which the follow-up of 5 years is complete.)*	
		6.5.2	What is the hazard ratio of revision due to infection for men compared to women, adjusted for:<br>- BMI<br>- Age<br>- ASA score (I, II, III, IV)<br>- Type of fixation (cemented or non-cemented)<br>- Type of prosthesis (Reversed arthroplasty, total anatomical arthroplasty or hemiarthroplasty)<br>- The above combined (add which you've included)<br><br>*(Provide a hazard ratio with standard error)*	*BMI*
				Age
				ASA score
				Type of fixation
				Type of prosthesis
				Combined
	6.6	Do you have data on revision rate due to infection of primary shoulder arthroplasties for osteoarthritis at 10 years?		If not, proceed to question 7<br>If yes, proceed to question 6.6.1
		6.6.1	What is the risk of revision due to infection for men compared to women?<br>*(Provide a hazard ratio with standard error. Also provide #men and*	

			#women of which the follow-up of 10 years is complete.)	
		6.6.2	What is the hazard ratio of revision due to infection for men compared to women, adjusted for:<br>- BMI<br>- Age<br>- ASA score (I, II, III, IV)<br>- Type of fixation (cemented or non-cemented)<br>- Type of prosthesis (Reversed arthroplasty, total anatomical arthroplasty or hemiarthroplasty)<br>- The above combined (add which you've included)<br><br>*(Provide a hazard ratio with standard error)*	*BMI*<br>*Age*<br>*ASA score*<br>*Type of fixation*<br>*Type of prosthesis*<br>*Combined*

	7	Have you recorded details of revision rates due to infection in primary ankle arthroplasty for osteoarthritis?		If not, this is the end of the survey<br>If yes, proceed to question 7.1
	7.1	What is the mean age of all primary ankle arthroplasties for osteoarthritis in your registry?<br>*(provide mean age and standard deviation)*		
	7.2	What is the gender distribution of all primary ankle arthroplasties for osteoarthritis in your registry?<br>*(provide in numbers with percentage)*		
	7.3	What is the start of data collection?		
	7.4	Do you have data on revision rate due to infection of primary ankle arthroplasties for osteoarthritis at 1 year?		If not, proceed to question 7.5<br>If yes, proceed to question 7.4.1
		7.4.1	What is the risk of revision due to infection for men compared to women?<br>*(Provide a hazard ratio with standard error. Also provide #men and #women of which the follow-up of 1 year is complete.)*	
		7.4.2	What is the hazard ratio of revision due to infection for men compared to women, adjusted for:<br>- BMI<br>- Age<br>- ASA score (I, II, III, IV)	*BMI*<br>*Age*

		- Type of fixation (cemented or non-cemented)<br>- Type of prosthesis (Total arthroplasty or hemiarthroplasty)<br>- The above combined (add which you've included)<br><br>*(Provide a hazard ratio with standard error)*	*ASA score*<br><br>*Type of fixation*<br><br>*Type of prosthesis*<br><br>*Combined*
7.5	Do you have data on revision rate due to infection of primary ankle arthroplasties for osteoarthritis at 5 years?		If not, proceed to question 7.6<br>If yes, proceed to question 7.5.1
	7.5.1	What is the risk of revision due to infection for men compared to women?<br>*(Provide a hazard ratio with standard error. Also provide #men and #women of which the follow-up of 5 years is complete.)*	
	7.5.2	What is the hazard ratio of revision due to infection for men compared to women, adjusted for:<br>- BMI<br>- Age<br>- ASA score (I, II, III, IV)<br>- Type of fixation (cemented or non-cemented)<br>- Type of prosthesis (Total arthroplasty or hemiarthroplasty)<br>- The above combined (add which you've included)<br><br>*(Provide a hazard ratio with standard error)*	*BMI*<br><br>*Age*<br><br>*ASA score*<br><br>*Type of fixation*<br><br>*Type of prosthesis*<br><br>*Combined*
7.6	Do you have data on revision rate due to infection of primary ankle arthroplasties for osteoarthritis at 10 years?		If not, this is the end of the survey<br>If yes, proceed to question 7.6.1
	7.6.1	What is the risk of revision due to infection for men compared to women?<br>*(Provide a hazard ratio with standard error. Also provide #men and*	

			#women of which the follow-up of 10 years is complete.)	
		7.6.2	What is the hazard ratio of revision due to infection for men compared to women, adjusted for:<br>- BMI<br>- Age<br>- ASA score (I, II, III, IV)<br>- Type of fixation (cemented or non-cemented)<br>- Type of prosthesis (Total arthroplasty or hemiarthroplasty)<br>- The above combined (add which you've included)<br><br>*(Provide a hazard ratio with standard error)*	*BMI*
				Age
				ASA score
				Type of fixation
				Type of prosthesis
				Combined
